# CFD–PBM Simulation for Continuous Hydrothermal Flow Synthesis of Zirconia Nanoparticles in a Confined Impinging Jet Reactor

**DOI:** 10.3390/ma16093421

**Published:** 2023-04-27

**Authors:** Qingyun Li, Zihua Wang, Xuezhong Wang

**Affiliations:** 1 School of Materials and Environment, Beijing Institute of Technology, Zhuhai 519088, China; 01010@bitzh.edu.cn; 2 School of Chemistry and Chemical Engineering, South China University of Technology, Guangzhou 510641, China; 3 Pharmaceutical and Crystallization Systems Engineering Group, Beijing Key Laboratory of Enze Biomass and Fine Chemicals, School of Chemical Engineering, Beijing Institute of Petrochemical Technology, Beijing 102617, China

**Keywords:** continuous hydrothermal flow synthesis, confined impinging jet mixing reactor, zirconia nanoparticles, population balance model, computational fluid dynamics

## Abstract

Computational fluid dynamics (CFD) and population balance models (PBM) were coupled together for the first time to simulate the synthesis of zirconia nanoparticles in a continuous hydrothermal flow synthesis (CHFS) system with a self-designed confined impinging jet mixing (CJM) reactor. The hydrodynamic and thermodynamic behaviors within the CJM reactor strongly influenced the formation of the ZrO_2_ nanoparticles. Crucial parameters, such as velocities, temperatures, mixing conditions, and reaction rates, were analyzed under various supercritical conditions. Temperature and velocity measurements as functions of distance were also investigated. Normal particle size distribution (PSD) patterns were observed in all cases. The mean particle sizes in this study were calculated and compared using PBM aggregation analysis.

## 1. Introduction

Due to the outstanding properties of zirconia, such as a high melting point, high resistivity, and low thermal expansion coefficient [1], ZrO_2_ nanoparticles have attracted much attention with extensive applications [2,3,4,5,6]. However, traditional preparation methods, such as sol–gel [7], hydrothermal [8], coprecipitation process [9], and combustion synthesis [10], are often energy- and time-consuming. The use of organic solvents is harmful to the environment as well. Take the sol–gel method for example: the preparation process involves the use of metallic alkoxides, various solvents, catalysts, and additives. Usually, the whole sol–gel process takes a long time [11,12]. There are many micropores in the gel, and, in the drying process, a great deal of gas and organic matter will escape and produce shrinkage, resulting in uneven preparation of nanoparticles.

A continuous hydrothermal flow synthesis (CHFS) system has been previously developed and investigated to overcome the limitations of hydrothermal batch processes [13]. In CHFS processes, a high-pressure liquid phase pump is used to send metal salt solution at normal temperature and preheated deionized water into the reactor for mixing. The temperature of deionized water after preheating is maintained above 374 °C, and the pressure of the whole system is maintained above 22.4 MPa by back pressure valve. At this temperature and pressure condition, water is in a supercritical state (the critical point of water is 374 °C, 22.1 MPa). Nanoparticles are produced rapidly in a very short residence time (usually a few seconds to a few minutes) when a salt solution at room temperature is mixed with supercritical water. Instead of slowly heating the solution, the CHFS system can reach extremely high temperatures in a few seconds. This is completed by mixing an aqueous solution of the precursor with a supercritical water (SCW) stream. The system offers a number of advantages during the synthesis of metal oxide nanoparticles [14]. First, this system is considered an environmentally friendly technology that uses supercritical water (SCW) as the reagent rather than organic solvents [15]. Second, because it is operated in a continuous mode in comparison with batch processes used in traditional hydrothermal methods, better control without batch-to-batch variation can be achieved. In addition, metal oxide nanoparticles can be produced in a much faster way, usually in seconds [14], due to the extreme conditions applied in the system. The obtained nanoparticles in water travel at a high flow rate throughout the process. Thus, crystalline growth and particle agglomeration are limited due to the low concentration suspension [16], which in turn increases the production of nanoparticles [17]. In summary, the process of preparing nanoparticles by CHFS does not require the use of protective atmosphere and organic solvent, and the process repeatability is good. The morphology of prepared particles is uniform. CHFS is an ideal preparation method for nanoparticles.

To improve the CHFS performance and, hence, transfer the process from the laboratory to the industrial scale, successful design and development of the reactor is important to ensure that the obtained material is of high purity and consists of ultrafine nanoparticles. Moreover, an optimized strategy for the in-depth understanding of the reactor, including both hydrodynamic and thermodynamic properties, is critical during the synthesis stage. However, this often consumes considerable time due to the huge experimental workloads required to design an alternative reactor. Therefore, computational fluid dynamics (CFD) has been extensively employed and developed as a modeling tool for CHFS analysis [18,19,20,21].

Ma et al. [22] conducted a CFD analysis and compared the fluid flows, heat transfers, and mixing behaviors between counter-current and confined impinging jet mixer (CJM) reactors. The research findings showed that mixing in the CJM reactor was much faster than that in the counter-current reactor. A numerical model for mixing and heat transfer analysis was also developed to scale up the process via temperature validations inside the reactor [23]. Compared with the other reactors [19,24,25,26,27], the CJM reactor showed outstanding performance in generating effective fluid mixing and heat transfer [23,28,29]. Thus, this reactor favored the formation of nanoparticles.

It Is well known that the particle size distribution (PSD) plays an important role in product quality due to the mixing conditions of the reactor, primary nucleation, crystalline growth, and particle aggregation. The population balance model (PBM), first proposed by Hulburt and Katz, is a general method to describe the particle size distribution of the dispersed phase in a multiphase flow system, which can well describe the particle growth and aggregation effect in multiphase flow [30]. The PBM has been widely used as a modeling tool for CHFS analysis to estimate the dynamic evolution of the obtained PSD as a function of operating conditions [20,31].

Winterer et al. [32] prepared nano- and micron zirconia powders and studied them via in situ high-pressure X-ray diffraction. Becker et al. [33] synthesized zirconia nanoparticles with mean particle sizes below 10 nm in near-critical and supercritical water, as well as supercritical isopropyl alcohol, in a continuous flow reactor. Masoodiyeh et al. employed PBM numerical simulation to predict the PSD of zirconia in a supercritical water hydrothermal synthesis process. The simulations were analyzed using batch reactors, including nuclear and crystal growth with/without aggregation [34]. Liu et al. [35] reviewed the characteristics and mechanisms of dissolution, crystallization, and growth of nano-zirconia during sub-/supercritical hydrothermal synthesis. According to the literature, numerical and experimental PSD investigations for the synthesis of ZrO_2_ nanoparticles using the CHFS–CJM system have not yet been reported. This work was aimed at addressing these shortcomings in the literature.

In our previous work, we combined CFD and PBM for simulation studies and compared the performance of a joint model of aggregation and surface growth with a model that only included surface growth as the sole mechanism for particle size enlargement [36]. In this paper, the CFD–PBM model was built to simulate the CHFS–CJM process under various supercritical conditions. The CFD simulation of flow field is more comprehensive and detailed, including temperature, velocity, density distribution, turbulent viscosity, Prandtl number, precursor mass fraction distribution, and second-phase volume fraction. More importantly, the reaction model is directly embedded into the CFD model to simulate the reaction rate distribution for the first time. The PBM model was developed and compared based on crystalline growth and aggregation. To verify the accuracy of the modeling approach employed, the predicted PSDs with/without aggregation were compared with the experimental results.

## 2. Experimental and Modeling

### 2.1. CHFS–CJM System Conditions

**Figure 1:**
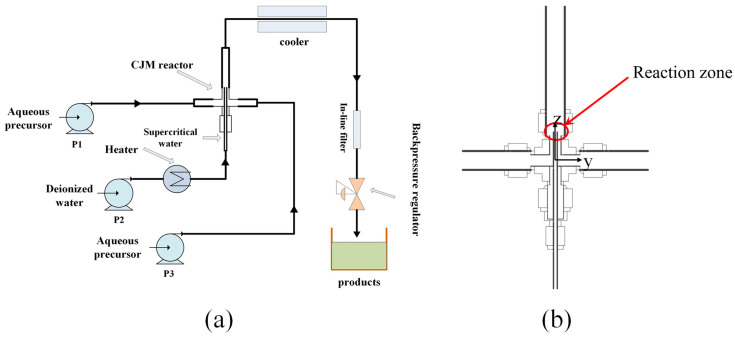
(**a**) Flow diagram of the CHFS system and (**b**) a schematic diagram of the CJM reactor.

The CHFS coupled with a CJM reactor, as shown in Figure 1a, was designed with an inner tube (Di = 0.99 mm) inserted into an outer tube (Di = 4.57 mm) with two horizontal feeders. The SCW mixed with the ambient precursor in a coaxial arrangement in the reaction zone, as shown in Figure 1b. Aqueous solutions (0.1 M) prepared with zirconyl nitrate (ZrO(NO_3_)_2_•xH_2_O) or zirconyl chloride octahydrate (ZrOCl_2_) were horizontally injected into the reactor via pumps P1 and P3, respectively. A pH of 9–10 was obtained by a KOH solution (0.5 M) at room temperature. The precursor flow rate was set to 5 mL/min. The SCW at 673 or 723 K was vertically pumped into the reactor via pump P2 at a constant flow rate of 10 mL/min. The system pressure was maintained at 24 ± 0.1 MPa. The obtained suspension traveled upwards before rapid cooling and harvest. The process details of all samples are shown in Table 1.

### 2.2. Characterization Methods

X-ray diffraction analysis (XRD, X’pert Powder PANalytical, Almelo, Netherlands) was carried out with 2θ = 5–90° using Cu Kα (λ = 1.541 Å) radiation under ambient conditions. The measurement uncertainty of diffraction angle indication error is less than one third of the indication error limit. The scanning electron microscopy imaging (SEM) (TESCAN, Brno, Czech Republic) of the ZrO_2_ nanoparticles was prepared with a TESCAN MIRA LMS Field Emission Scanning Electron Microscope. Transmission electron microscopy (TEM, JEOL JEM-2100F, Tokyo, Japan) was employed with the particle size distribution (PSD) measured by the ImageJ V1.8.0.112 package.

### 2.3. CFD–PBM Model Development

The zirconia formation reactions were described by a hydrolysis step and a dehydration step, as shown below [37].

(1) $${\mathrm{ZrO}\;+\;3H}_{2}O\;\to \;{\;\mathrm{Zr}(\mathrm{OH})}_{4}\;+\;{2H}^{+}{+2X\;X\;=\;\mathrm{Cl}}^{-}{\;\mathrm{or}\;\mathrm{NO}}_{3}{}^{-}$$

(2) $${\mathrm{Zr}(\mathrm{OH})}_{4}\;\to \;{\mathrm{ZrO}}_{2}\;+\;{2H}_{2}O$$

in conventional hydrothermal process, where synthesis happens at lower temperatures (273–473 K), it is believed that the hydrolysis step is fast and almost instantaneously results in precipitation of gel-like M(OH)_x_ hydroxide solids. The dehydration step, on the other hand, is relatively slow due to the chemical nature of hydroxides; therefore, the whole process can take hours or even days to complete. In supercritical condition, the dehydration rate is much enhanced by the increasing temperature, which substantially contributes to a much shorter reaction time of less than 2 s for both reaction steps [38]. As a result, the pre-occurred gel-like metal hydroxide would not have enough time to grow but immediately dehydrated to form metal oxides.

The CFD fluid dynamics model was combined with the PBM model to predict the size distribution of zirconia by interacting with the species transport equation for pre-nucleation concentration measurements, the reaction equation for metal oxide formulation reactions, and user-defined functions for nucleation, growth, and aggregation of crystal. The calculation domain of CJM reactor was generated by Gambit 2.4. The reactor was discretized with 3.0 × 10^5^ tetrahedral unit consisting of stainless steel inner and outer tubes connected with two horizontal feeders, as shown in Figure 1.

The thermodynamic properties of water were calculated using the IAPWS formulation 1995 [39]. In this work, the complex IAPWS formulation was represented by several polynomial equations by piece-wise curve-fitting the thermo-physical chart of water at 24.1 MPa [29]. An example of these polynomial equations for thermal conductivity (κ) within a temperature range of 273–618 K is listed as:

*κ* = −0.267 + 4.61 × 10^−6^
*T* − 5.48 × 10^−6^
*T*^2^ (3)

note that the low precursor concentration (0.1 M) resulted in low ZrO_2_ nanoparticles in suspension (0.1% *w*/*w*) [16]. The metal species on the feeders were ignored. Thus, the properties of these metal salt solutions are identical to those of water. Note that the thermodynamic constants [31] were also obtained for this study.

A nonreacting hydrodynamic study was adopted by characterizing the flow regime as a liquid–solid multiphase by the Eulerian–Eulerian multiphase approach [40]. The primary phase was set as the mixture of the SCW, the precursor, and an intermediate liquid phase. However, the particulate phase was considered as a dispersed secondary phase. The volume was defined by the phase volume faction, as shown below.

(4) $$V_{i}\;=\;\int_{V}^{\;}\alpha_{i}dV$$

where $\alpha_{i}$ is the volume fraction of phase *i*:

(5) $$\sum_{i\;=\;1}^{n}\alpha_{i}\;=\;1$$

the Eulerian–Eulerian multiphase mode equations are listed below [41].

The continuity equation was written as

(6) $$\frac{\partial }{\partial t}\left(\alpha_{P}\rho_{P}\right)+\nabla \times \left(\alpha_{P}\rho_{P}\vec{v_{P}}\right)\;=\;\sum_{P\;=\;1}^{2}\left({\overset{•}{m}}_{PS}-{\overset{•}{m}}_{SP}\right)$$

where $\vec{v_{P}}$ is the velocity of the primary phase and ${\overset{•}{m}}_{PS}$ is the mass transfer from the primary phase to the secondary phase.

The momentum balance equation was written as

(7) $$\frac{\partial }{\partial t}(\alpha_{P}\rho_{P}\vec{v_{P}})\;+\;\nabla \;\times \;(\alpha_{P}\rho_{P}\vec{v_{P}}\vec{v_{P}})\;=\;{-\alpha }_{P}\nabla P\;+\;\nabla {\bar{\Gamma }}_{P}\;+\;\alpha_{P}\rho_{P}\vec{g}\;+\;\overset{2}{\underset{P\;=\;1}{\sum }}({\vec{F}}_{\mathrm{vm},P}\;+\;{\overset{•}{m}}_{SP}\vec{v_{SP}}\;-\;{\overset{•}{m}}_{PS}\vec{v_{PS}})\;+\;({\vec{F}}_{P}\;+\;{\vec{F}}_{\mathrm{lift},P}\;+\;{\vec{F}}_{\mathrm{in},P})$$

where $\vec{v_{\mathrm{PS}}}$ is the interphase velocity. If ${\overset{•}{m}}_{\mathrm{PS}}$ is greater than zero, $\vec{v_{\mathrm{PS}}}$ = $\vec{v_{P}}$; if ${\overset{•}{m}}_{\mathrm{PS}}$ is less than zero, $\vec{v_{\mathrm{PS}}}$ = $\vec{v_{S}}$ during the analysis.

(8) $${\bar{\Gamma }}_{P}{\;=\;\alpha }_{P}\mu_{P}(\nabla \vec{v_{P}}\;+\;\nabla \vec{{v_{P}}^{T}}{)\;+\;\alpha }_{P}{(\lambda }_{P}\;-\;\frac{2}{3}\mu_{P})\nabla \;\times \;\vec{v_{P}}\bar{I}$$

where ${\bar{\Gamma }}_{P}$ is the primary phase stress–strain tensor, $\mu_{P}$ and $\lambda_{P}$ are the shear and bulk viscosities of the primary phase, $\vec{F_{P}}$ is the external body force, ${\vec{F}}_{\mathrm{lift},P}$ is the lift force, ${\vec{F}}_{\mathrm{vm},P}$ is the virtual mass force, ${\vec{F}}_{\mathrm{in},P}$ is the interaction force between phases, and P is the pressure shared by all phases.

The secondary phase was calculated by subtracting the primary phase because the total volume fraction was set to one. The energy balance was given by the standard energy equation, as shown below.

(9) $$\frac{\partial }{\partial t}(\rho E)\;+\;\nabla \;\times \;(\vec{v}(\rho E\;+\;P))\;=\;\nabla {\;\times \;(k}_{\mathrm{eff}}\nabla T\;-\;\sum_{j}h_{j}\vec{J_{j}}\;+\;({\bar{\tau }}_{\mathrm{eff}}\;\times \;\vec{v}))$$

where $k_{\mathrm{eff}}$ is the effective conductivity and $\vec{J_{j}}$ is the diffusion flux of species *j*.

For multiphase systems, turbulence modeling is actually complicated because of the additional momentum equations. The standard *k*-*ε* model was selected in this investigation. The turbulent kinetic energy *k* and its dissipation rate *ε* were solved using standard empirical constants [42].

(10) $$\frac{\partial }{\partial t}(\rho k)\;+\;\frac{\partial }{\partial x_{i}}{(\rho k\mu }_{i})\;=\;\frac{\partial }{\partial x_{j}}\left[\left(\mu \;+\;\frac{\mu_{t}}{\sigma_{k}}\right)\frac{\partial k}{\partial x_{j}}\right]{\;+\;G}_{k}{\;+\;G}_{b}{\;-\;\rho \varepsilon \;-\;\Upsilon }_{M}$$

(11) $$\frac{\partial }{\partial t}(\rho \varepsilon )\;+\;\frac{\partial }{\partial x_{i}}{(\rho k\mu }_{i})\;=\;\frac{\partial }{\partial x_{j}}\left[\left(\mu \;+\;\frac{\mu_{t}}{\sigma_{\varepsilon }}\right)\frac{\partial \varepsilon }{\partial x_{j}}\right]{\;+\;G}_{1\varepsilon }\frac{\varepsilon }{k}{(G}_{k}{+C}_{\varepsilon 1}G_{b}{)\;-\;C}_{\varepsilon 2}\rho$$

(12) $$\mu_{t}\;=\;0.09\rho \frac{k^{2}}{\varepsilon }$$

$G_{k}$ is the turbulent kinetic energy generated by the average velocity gradient, $G_{b}$ is the turbulent kinetic energy due to buoyancy, $\Upsilon_{M}$ represents the contribution of pulsatile expansion in the compressibility turbulent dissipation rate, and $\sigma_{k}$ and $\sigma_{\varepsilon }$ are the turbulent Prandtl numbers for *k* and *ε*. The four empirical constants, $\sigma_{k}$, $\sigma_{\varepsilon }$, $C_{\varepsilon 1}$, and $C_{\varepsilon 2}$, are 1, 1.3, 1.44, and 1.92, respectively.

The primary phase composition was determined by a hydrothermal reaction model. The volume/mass weighted mixing law was employed during calculation. The diffusion coefficient of the mixture was solved using a modified Chapman–Enskog equation dependent on temperature [43].

Since the hydrolysis reaction was considered the dominant step, the hydrothermal reaction could be simplified as A + sB → C. The composition was determined by

(13) $$\frac{\partial {\rho Y}_{i}}{\partial t}\;+\;\frac{\partial {\rho u}_{j}Y_{i}}{\partial x_{j}}\;=\;\frac{\partial }{\partial x_{i}}\left(\Gamma_{i,\mathrm{eff}}\frac{\partial Y_{i}}{\partial x_{j}}\right){\;+\;R}_{i}$$

*R_i_* is the net reaction rate, defined as

(14) $$R_{i}\;=\;\Gamma (\nu_{i,p}\;-\;\nu_{i,r})[\kappa_{f}\prod_{i\;=\;1}^{N}{\left(C_{i}\right)}^{\left(\eta_{i,r}\;+\;\eta_{i,p}\right)})]$$

where $\nu_{i,p}$ and $\nu_{i,r}$ represent the stoichiometric coefficients, $\eta_{i,p}$ and $\eta_{i,r}$ represent the rate exponents for the products and reactants, $C_{i}$ is the molar concentration, and $\kappa_{f}$ is the reaction rate constant calculated by the Arrhenius expression, as shown below:

(15) $$\kappa_{f}{\;=\;A}_{r}{T\;\times \;e}^{{(-E}_{r}/RT)}$$

where $A_{r}$ is the pre-exponential factor and $E_{r}$ is the activation energy [44].

To determine the PSD of the obtained ZrO_2_ nanoparticles produced by the CHFS–CJM process, a number density function was introduced with the PBM equation, as shown below.

(16) $$\frac{\partial }{\partial t}{(\rho }_{s}\alpha_{i})\;+\;\nabla {(\rho }_{s}\mu_{i}\alpha_{i})\;+\;\frac{\partial }{\partial V}(\frac{G_{v}\rho_{s}\alpha_{i}}{V}){\;=\;\rho }_{s}V_{i}{(B}_{\mathrm{ag},i}{\;-\;D}_{\mathrm{ag},i}{\;+\;B}_{\mathrm{br},i}{\;-\;D}_{\mathrm{br},i}{)\;+\;0}^{i}\rho_{s}V_{0}{\overset{•}{n}}_{0}$$

where *ρ_s_* is the density of the secondary phase, *α_i_* is the volume fraction, *V*_0_ is the volume of the smallest particle size, ${\overset{•}{n}}_{0}$(1/m^3^) − s is the nucleation rate, and *G*_*V*_ is the growth rate of particles.

Primary nucleation was the predominant mechanism for the nucleation rate under supersaturation conditions [24]. Classical homogenous nucleation theory [45] was applied in this investigation.

(17) $${\overset{\cdot }{n}}_{0}\;=\;\mathrm{Aexp}\left[-\frac{B}{{\left(\mathrm{lnS}\right)}^{2}}\right]$$

where *A* is the pre-exponential factor, *A* = (3–9) × 10^18^, *B* is the constant determined by the temperature and interfacial energy of the precursor solution, in this case, *B* = 100. *S* is the degree of supersaturation, which is a function of temperature and density of water.

The degree of supersaturation could be calculated using the concentration and solubility values of ZrO_2_. The kinetics of crystalline growth can be expressed as shown below.

(18) $$G_{v}{\;=\;k}_{g}{\left(S-1\right)}^{g}$$

where *k_g_* = 3 × 10^−10^ m·s^−1^ and *g* =1 are the kinetic constants.

The aggregation kernel was defined as a product of the frequency of collisions and the efficiency of aggregation. In this study, the free molecule model based on Brownian kernel function [46] is selected and the size effect in the process of particle collision is considered. For the submicron particle analysis, the Brownian kernel function [45,47] was selected in this investigation.

The PBM equations were combined with the CFD analysis using the Sauter mean diameter approach. The particle sizes were first expressed by the Sauter mean diameters and then converted into the length diameters. A comparison between the Sauter mean diameter *D*_3,2_ (i.e., the mean particle size based on the surface area) and the length mean diameter *D*_1,0_ (i.e., the mean particle size based on the particle diameter) was described, as shown below.

(19) $$D_{3,2}\;=\;\frac{1}{N}\times \frac{\sum_{i\;=\;1}^{N}{\mathrm{NiDi}}^{3}}{\sum_{i\;=\;1}^{N}{\mathrm{NiDi}}^{2}}$$

(20) $$D_{1,0}\;=\;\frac{1}{N}\times \sum_{i\;=\;1}^{N}\mathrm{NiDi}$$

where *D* is the particle diameter (nm) and *N* is the total number of particles.

The PSD simulation conditions were detailed in Cases 1–4, as shown in Table 1. The PBM equation is solved by homogeneous discretization. All fluid properties within the CJM reactor were solved using ANSYS Fluent 16.0. The steady-state flow method and the finite volume method were used to solve the equation numerically for the simulation. A standard SIMPLE pressure–velocity solver and a first-order upwind scheme are used to discrete the convective terms in the equation [19].

## 3. Results and Discussion

**Figure 2:**
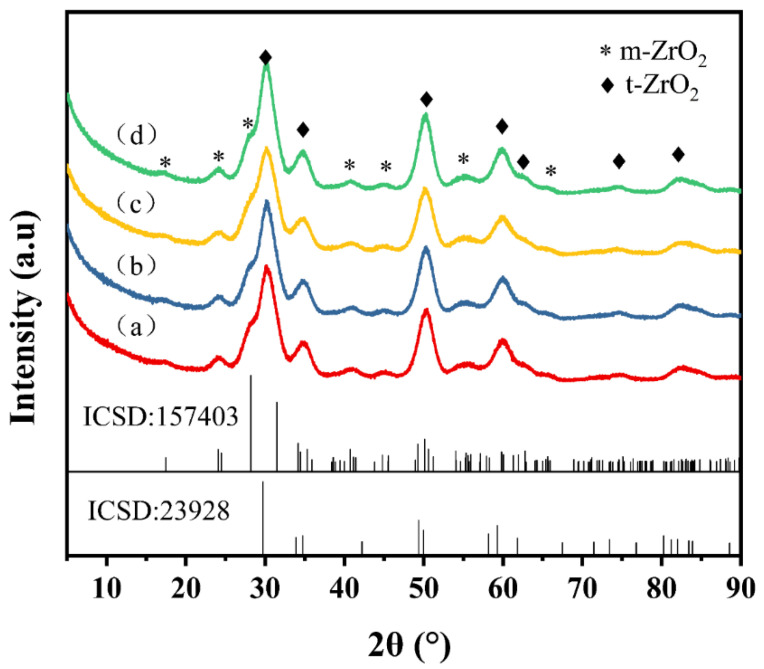
XRD patterns of samples synthesized: (**a**) case 1; (**b**) case 2; (**c**) case 3; (**d**) case 4.

The XRD results of the obtained particles are shown in Figure 2. The phase composition of the ZrO_2_ nanocrystals was clearly demonstrated by XRD patterns. The diffraction peaks of synthesized ZrO_2_ can be indexed as tetragonal (ICSD 23928) and monoclinic (ICSD 157403) phase. The main specific diffractive peaks appear at the 2θ values of 30.1°, 34.7°, 50.1°, 60.0°, 74.5°, and 82.1°, which are in good agreement with the reference pattern of tetragonal zirconia (t-ZrO_2_). Several broad and low-intensity diffraction peaks at the 2θ values of 17.2°, 24.0°, 40.7°, 45.1°, 55.2°, and 65.8° can be observed, indicating monoclinic phase (m-ZrO_2_). As can be seen from the figure, the results of all experiment cases are highly consistent.

**Figure 3:**
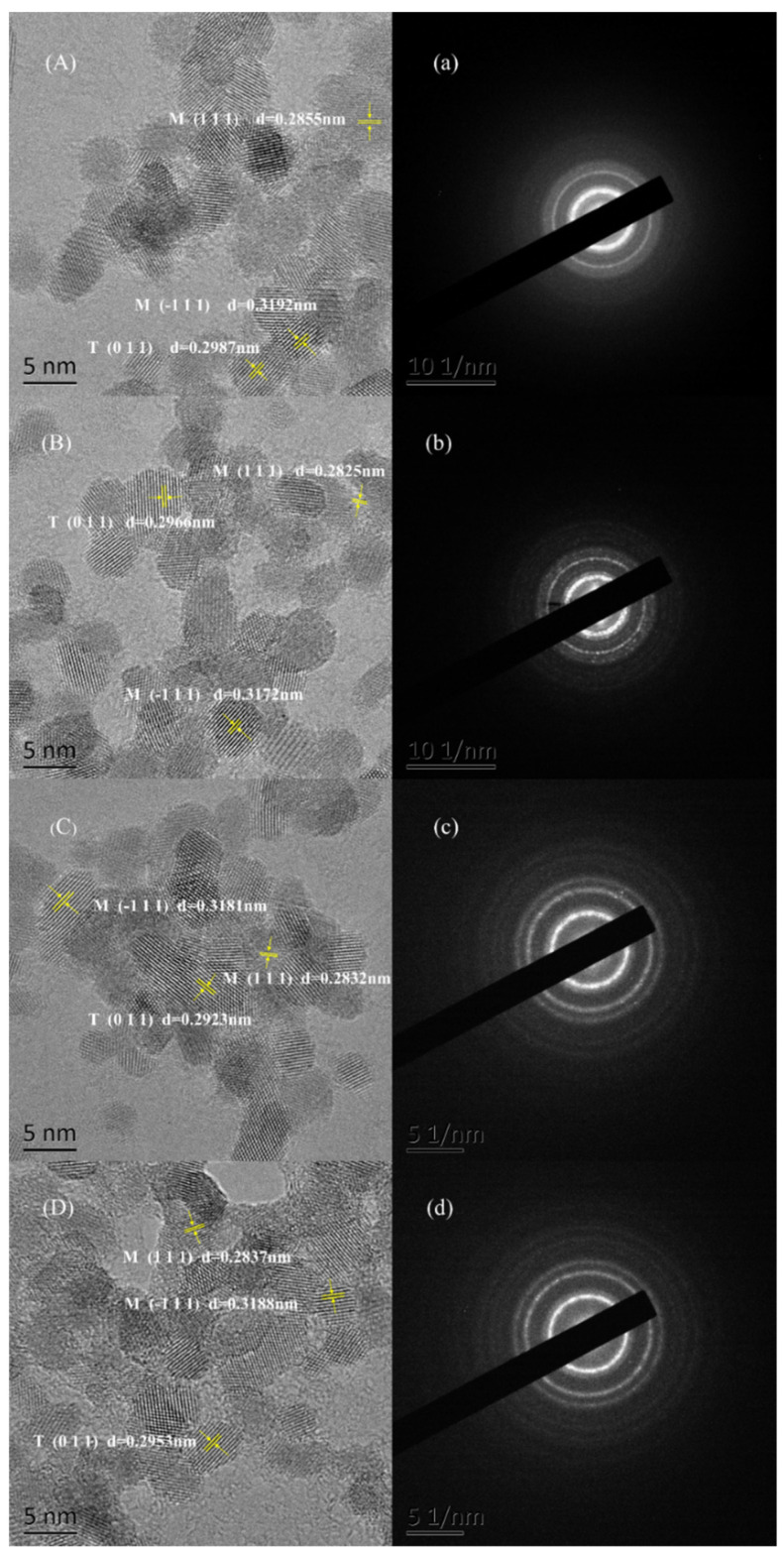
(**A**–**D**) HR-TEM images and (**a**–**d**) respective SAED patterns of the ZrO_2_ nanoparticles: (**A**,**a**) for case 1; (**B**,**b**) for case 2; (**C**,**c**) for case 3; (**D**,**d**) for case 4. Note that the tetragonal (T) or monoclinic (M) nanoparticles were indexed in the images.

The HR-TEM images of zirconia nanoparticles and nanosheets are shown in Figure 3A–D. It can be seen from the figures that all nano-ZrO_2_ samples have uniform particle shape and narrow particle size distribution, ranging from 3 nm to 6 nm. The (–1 1 1) crystal faces and (1 1 1) crystal faces obtained from HR-TEM images belong to monoclinic phase ZrO2, while the (0 1 1) crystal faces belong to tetragonal phase ZrO_2_. The selected area electron diffraction (SAED) images of zirconia are shown in Figure 3a–d. A large number of diffraction rings in the ED pattern confirm the polycrystalline nature of spherical particles, which is in agreement with the XRD patterns.

**Figure 4:**
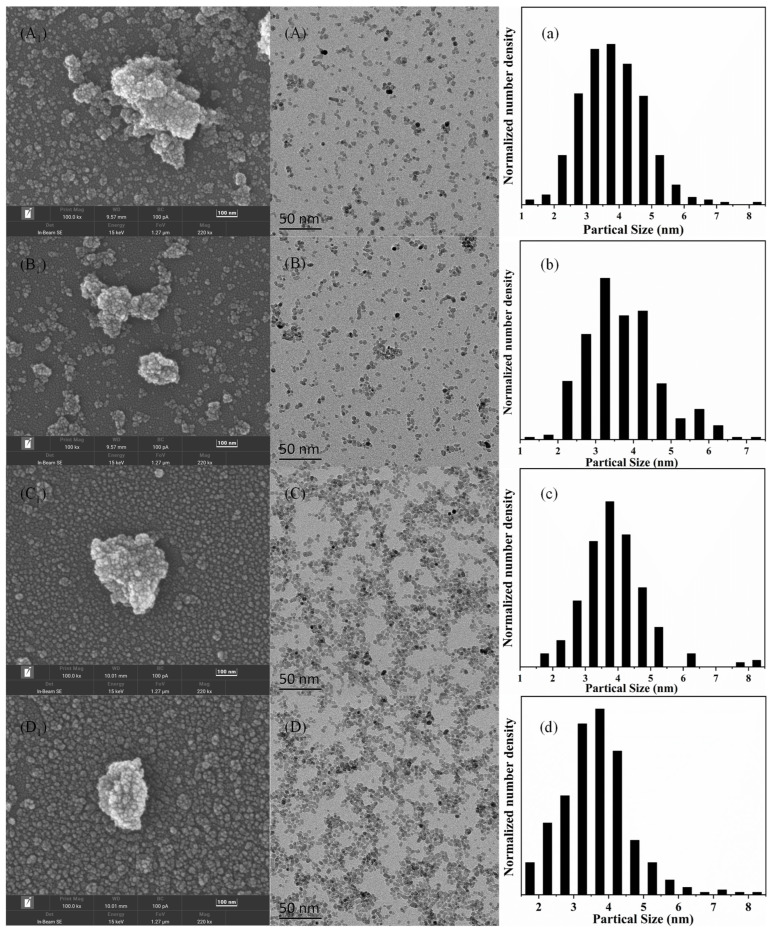
(**A_1_**–**D_1_**) SEM images, (**A**–**D**) TEM images, and (**a**–**d**) respective PSD analysis of the ZrO_2_ nanoparticles: (**A_1_**,**A**,**a**) for case 1; (**B_1_**,**B**,**b**) for case 2; (**C_1_**,**C**,**c**) for case 3; (**D_1_**,**D**,**d**) for case 4.

The morphology, size, and aggregation of the obtained nanoparticles are shown in Figure 4. The SEM micrographs of all the samples are presented in Figure 4A_1_–D_1_. Similar morphology is observed in all cases, showing that the samples were agglomerated. Nanoparticles tend to agglomerate due to the small particle size and high surface energy, resulting in particle non-steady-state thermodynamics. According to the TEM analysis, highly crystallized ZrO_2_ nanoparticles were observed in all samples with uniform size and shape distributions, as shown in Figure 4A–D. It is worth noting that, compared with the SEM images, the TEM images showed less agglomeration of nanoparticles, which may be determined by the characteristics of TEM sample preparation, that is, smaller sample size and more fully dispersed samples. The particle size distribution obtained from TEM micrographs is shown in Figure 4a–d. A Gaussian profile was used to fit to the size distribution of the nanoparticles. It is found that the mean particle size is about 3–5 nm in all samples.

**Figure 5:**
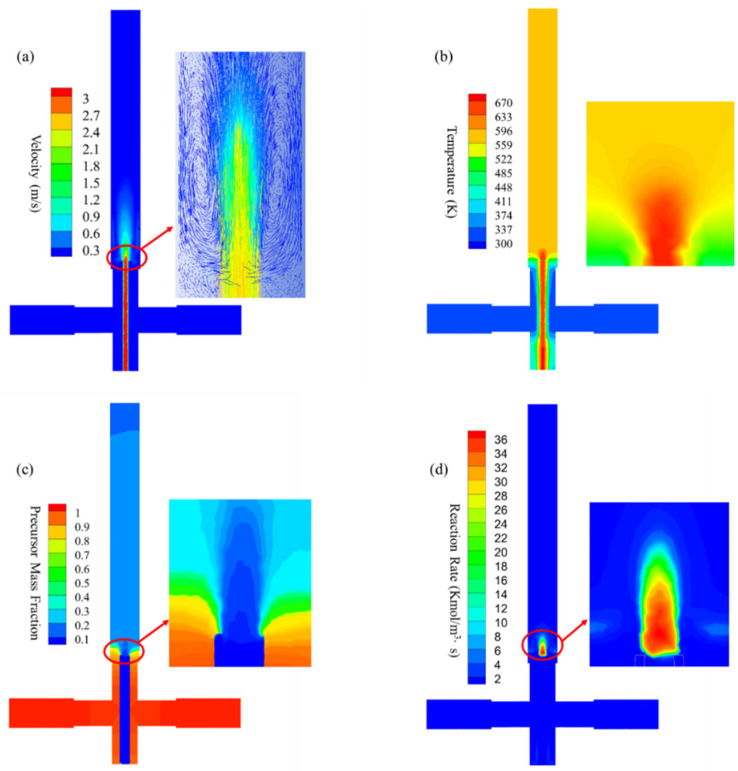
CFD simulation of the reactor: (**a**) velocity distribution, (**b**) temperature distribution, (**c**) precursor mass fraction distribution, and (**d**) reaction rate in the reaction zone during ZrO_2_ nanoparticles production.

A co-current flow pattern was observed in the reaction zone, as shown in Figure 5a, which induced recirculation phenomena with high feeding rates (10 and 5 mL/min) during operation. The temperature quickly reached equilibrium at approximately 600 K during the mixing process, as shown in Figure 5b. Under supercritical conditions, the product solubility was reduced, thereby favoring particle nucleation. As shown in Figure 5c, a high degree of mixing was observed with no back-mixing phenomenon in the reaction zone. The precursor mass fraction rapidly decreased and reached equilibrium at approximately 20% due to the dilution and reaction processes applied by the SCW. As shown in Figure 4d, the highest reaction rate distribution was measured to be approximately 35 Kmol/m^3^-s at the center of the reactor. Note that the reaction rate was calculated based on the formation of zirconium hydroxide. The reaction rate distribution decreased and reached approximately 2 Kmol/m^3^∙s, as shown in the insert of Figure 5d. This allowed the nucleation of ZrO_2_ nanoparticles and, hence, prevented particle coarsening during the process.

**Figure 6:**
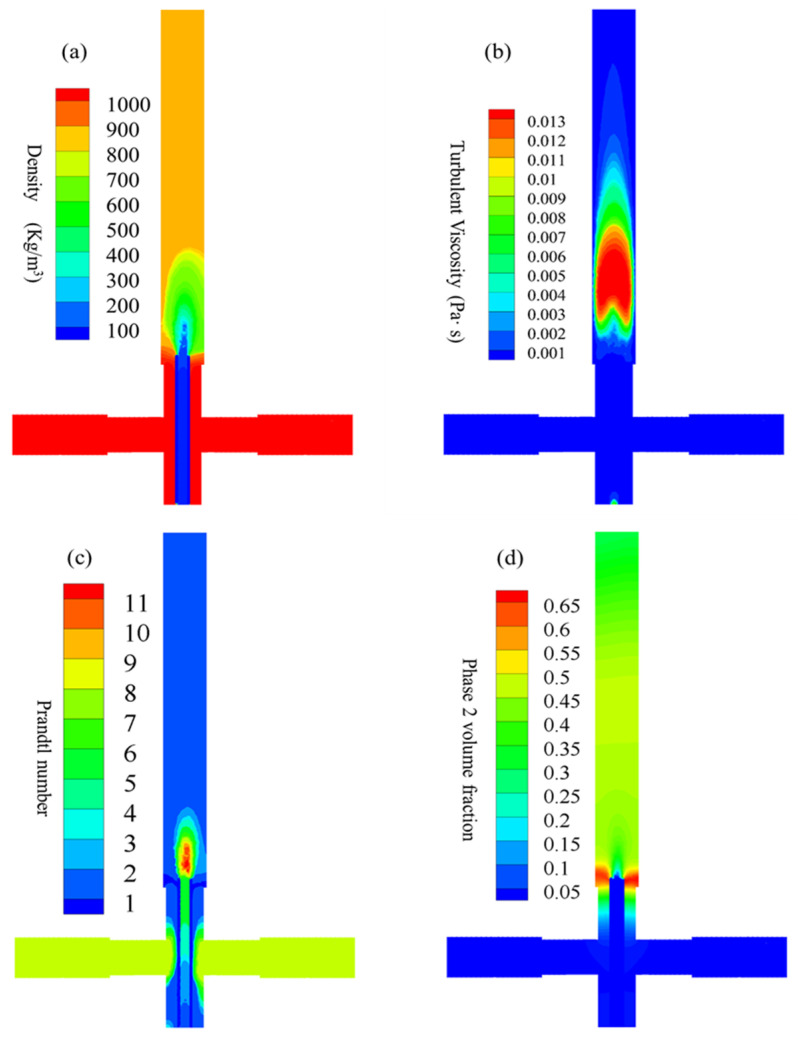
CFD simulation of the reactor: (**a**) density distribution, (**b**) turbulent viscosity, (**c**) Prandtl number, and (**d**) 2nd-phase volume fraction in the reaction zone during ZrO_2_ nanoparticles production.

Figure 6a shows the density distribution of the mixing behavior between the SCW and the precursor solution. Note that the densities of the precursor solution were set as room temperature water in this investigation. Under supercritical conditions, the density decreased sharply due to the dilution of the SCW. A homogeneous mixture was obtained as the density gradually increased in the reaction zone, as shown in Figure 6a. Comparable behavior was observed in the turbulent viscosity analysis, as shown in Figure 6b. The turbulent viscosity was much higher (0.013 Pa·S) at the center in comparison with the outside areas. Note that a typical shape (see Figure 6b red area) was found due to the recirculation phenomenon shown in the insert of Figure 5a. Figure 6c shows the Prandtl number distribution, which is directly related to the physical properties of the fluid mixture during the convective heat transfer process. The initial Prandtl number was calculated as approximately 8 when the precursor was injected into the reactor. According to the hydrothermal process, zirconium hydroxide was initially formed with rapid transformation into ZrO_2_ nanoparticles. Therefore, a high Prandtl number (approximately 10–11) was measured in the reaction zone. As the suspension continuously traveled upwards for cooling and harvest, the Prandtl number decreased, as shown in Figure 6c. This was further confirmed by the second-phase volume fraction analysis, as shown in Figure 6d. Note that the first-phase volume fraction was defined as the pre-nucleation zirconia and the second-phase as post-nucleation in this investigation. As shown in Figure 6d, some of the ZrO_2_ nuclei accumulated in the recirculation areas (see Figure 5a insert), while the rest were distributed in the reaction zone.

Excellent performance, such as rapid mixing phenomenon and homogeneous distributions, was found in this investigation. Thus, nucleation was ensured while preventing particle enlargement during ZrO_2_ nanoparticle synthesis using the CHFS–CJM system. Due to the high flow rate obtained in the process, hydrothermal reactions took place within seconds and only in the reaction zone. Rapid cooling ensured that the obtained samples were ultrafine ZrO_2_ nanoparticles. Due to the limitations of the CFD analysis, no obvious differences were found in the cloud diagrams (see Figure 5 and Figure 6) when using different precursors or temperatures, as shown in Table 1. The CFD results presented in this investigation were obtained in Case 3, as shown in Figure 5 and Figure 6.

**Figure 7:**
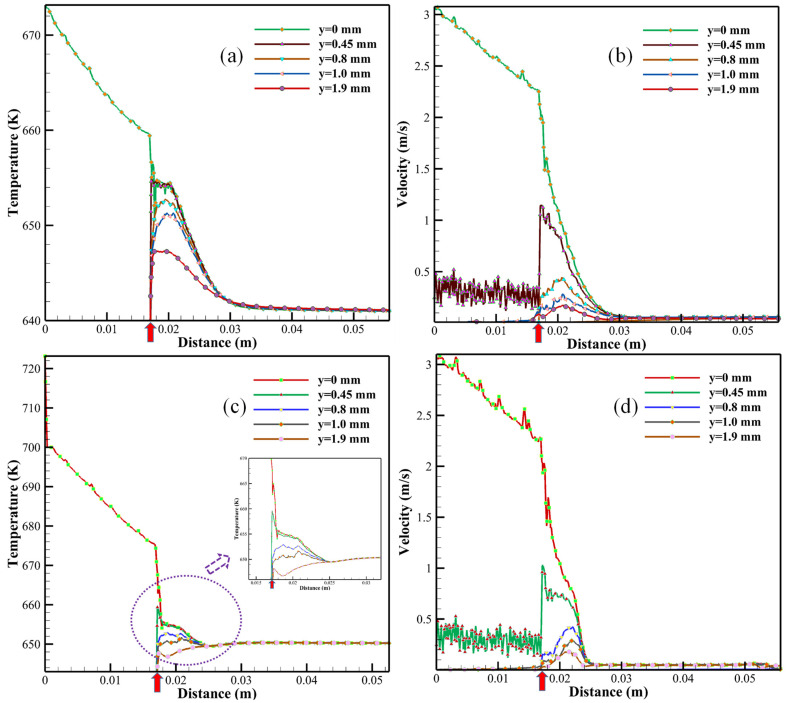
Temperature and/or velocity as a function of distance along the y-axis in the reaction zone where (**a**,**b**) were obtained in Case 3 in comparison with (**c**,**d**) in Case 4. Note that the red arrow indicates the outlet of the inner tube along the z-axis.

The changes in temperatures and velocities were analyzed as a function of distances along the y-axis (see Figure 1b), where y = 0, 0.45, 0.8, 1.0, and 1.9 mm inside the CJM reactor at 673 and 723 K using the ZrO(NO_3_)_2_•H_2_O precursor, as shown in Figure 7. According to the CFD analysis in Case 3, as shown in Figure 7a, the initial temperature was measured to be 673 K along the central line of the reactor where y = 0 mm. The temperature decreased slightly to approximately 659 K (see the red arrow in Figure 7a) before the SCW was injected into the reaction zone at the outlet of the inner tube. Note that the temperature dropped sharply from 659 to 654 K, possibly due to the mixing phenomenon between the SCW and the room temperature precursor solution inside the CJM reactor. Rapid hydrolysis and dehydration reactions resulted in the formation of ZrO_2_ nanoparticles; thus, a further temperature decrease was observed, as shown in Figure 7a. Note that the obtained final temperature (approximately 641 K) was higher than the critical temperature of 640 K, which ensured ZrO_2_ crystalline growth and possible aggregation during operation. As the temperature profiles moved from the central line (y = 0 mm) to the outside of the reaction zone (y = 0.45, 0.8, 1.0, and 1.9 mm), fewer temperature differences were observed, as shown in Figure 7a, due to the temperature distribution phenomenon shown in Figure 5b. Note that, between approximately 0.017 and 0.032 m in the reaction zone, the temperature profiles, especially at y = 0.8 and/or 1.0 mm, were increased and then reduced, as shown in Figure 7a, possibly due to the high-temperature SCW being injected into the reaction zone. According to the flow velocity analysis along the central line (y = 0 mm), the initial rate was measured as 3.08 m/s, as shown in Figure 7b. The flow velocity decreased and was measured as approximately 2.25 m/s at the outlet of the inner tube (see the red arrow in Figure 7b). The flow velocity continuously decreased, and, hence, the obtained ZrO_2_ nanoparticles traveled through the harvesting process. This matched with the velocity distribution analysis (see Figure 5a) as a high flow rate was observed in the CJM reactor. At y = 0.45 mm, an unstable velocity profile was observed before the outlet of the inner tube, as shown in Figure 7b. The precursor solutions were added into the CJM reactor via two horizontal feeders, as shown in Figure 1. The flow rate increased to approximately 1.15 m/s as the SCW and the precursor mixed together, as shown in Figure 7b. According to the temperature and velocity profiles in Case 4 at 723 K, comparable results were observed, as shown in Figure 7c,d. Small temperature and/or velocity fluctuations were observed, possibly due to the turbulence phenomenon obtained in Cases 3 and 4. No obvious differences were obtained by using different precursors (see Table 1) due to the low concentrations set in the CFD analysis. The results calculated in Cases 3 and 4 were presented in this investigation only, as shown in Figure 7.

To understand the particle size distributions for the ZrO_2_ nanoparticles obtained under various supercritical hydrothermal conditions, as shown in Table 1, the CFD numerical analysis was coupled with the PBM investigation using no-aggregation mode (i.e., surface growth mode) and aggregation mode. Normalized calculated PSDs were compared with the experimental results in Figure 7a–d in the form of particle size number density. The experiment PSD data were determined by manually measuring around 200 particles using ImageJ.

**Figure 8:**
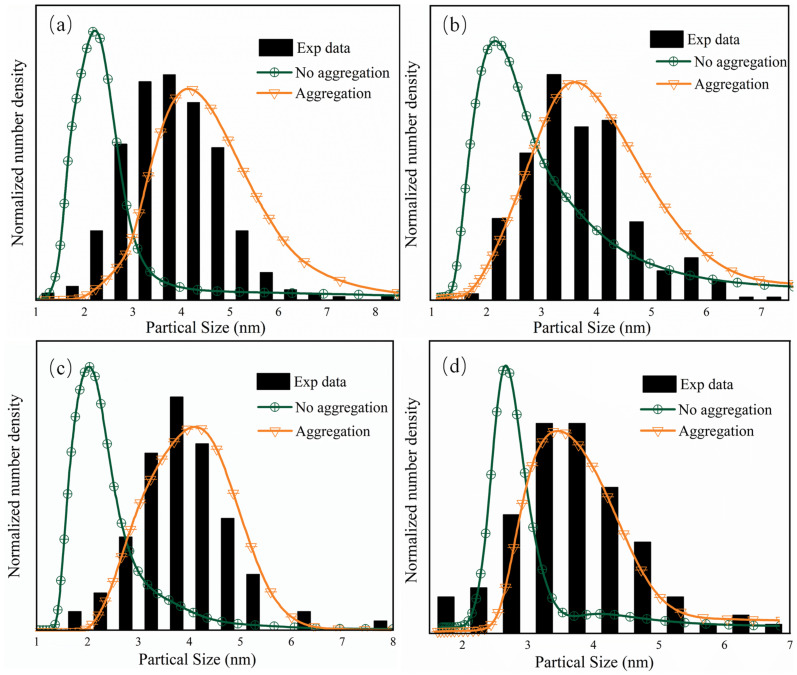
PSD comparison of the ZrO_2_ nanoparticles obtained under various conditions: (**a**) Case 1, (**b**) Case 2, (**c**) Case 3, and (**d**) Case 4.

As shown in Figure 8, the predicted PSD without aggregation was narrow, which showed that the mean particle size is about 2 nm, while the predicted PSD with aggregation showed that mean particle size is 3–5 nm, which is in good accordance with the TEM experimental results. This result suggests that the prepared nanoparticles were generally formed by the aggregation of crystal nuclei as well as surface growth. Moreover, the particle sizes correspond well to the crystallite sizes determined by XRD, which confirms that the particles are highly crystalline.

## 4. Conclusions

In this study, CFD–PBM analysis was successfully applied during the ZrO_2_ nanoparticles synthesis process using the CHFS–CJM system. The hydrodynamic and thermodynamic variables, including velocities, temperatures, and mixing behaviors, were simulated under different supercritical conditions. The PSD patterns obtained in all cases exhibited normal distributions by either surface growth or aggregation analysis. Particle aggregation was found to be an important parameter during the PSD simulation and prediction analysis. This was because the particle size enlargement was not only due to nucleation and crystalline growth but was also directly related to particle interactions.

Validation of the CFD–PBM model has been addressed using a simple and efficient methodology, evaluating all aspects of the flow physics and the behavior of a model reaction inside the reactor. However, as demonstrated in the paper, the model cannot accurately predict the particle agglomeration shown in the SEM image. Further research is needed to fully understand the relationship between kinetics and fluid dynamics in supercritical hydrothermal reactors.

## Figures and Tables

**Table 1:** CHFS operating conditions for the production of ZrO_2_ nanoparticles.

Samples	Precursors	Flow Rate (mL/min)	SCW Temperature (K)
Case 1	ZrOCl_2_	5	673
Case 2	ZrOCl_2_	5	723
Case 3	ZrO(NO_3_)_2_	5	673
Case 4	ZrO(NO_3_)_2_	5	723

## Data Availability

Not applicable.
